# Implementation of a Global Treatment Budget in Psychiatric Departments in Germany—Results and Critical Factors for Success From the Staff Perspective

**DOI:** 10.3389/fpsyt.2020.00610

**Published:** 2020-07-31

**Authors:** Sonja Indefrey, Bernard Braun, Sebastian von Peter, Andreas Bechdolf, Thomas Birker, Annette Duve, Olaf Hardt, Philip Heiser, Kerit Hojes, Burkhard Rehr, Harald Scherk, Anna Christina Schulz-Du Bois, Bettina Wilms, Martin Heinze

**Affiliations:** ^1^ Department of Psychiatry and Psychotherapy, Charité University Medicine Berlin, Berlin, Germany; ^2^ SOCIUM Research Center, University of Bremen, Bremen, Germany; ^3^ Department of Psychiatry and Psychotherapy, Brandenburg Medical School Theodor Fontane, Immanuel Klinik Rüdersdorf, Rüdersdorf, Germany; ^4^ Department of Psychiatry and Psychotherapy, Vivantes Kliniken am Urban und im Friedrichshain, Berlin, Germany; ^5^ Department for Psychiatry, Psychotherapy and Psychosomatic Medicine, Westküstenklinikum Heide, Heide, Germany; ^6^ Department of Child and Adolescent Psychiatry, Vitos Klinikum Riedstadt, Riedstadt, Germany; ^7^ Department of Psychiatry and Psychotherapy, Vivantes Klinikum Neukölln, Berlin, Germany; ^8^ Department of Child and Adolescent Psychiatry, Südharz Klinikum Nordhausen, Nordhausen, Germany; ^9^ Department of Psychiatry and Psychotherapy, Südharz Klinikum Nordhausen, Nordhausen, Germany; ^10^ Department of Psychiatry and Psychotherapy, Psychiatrische Klinik Lüneburg, Lüneburg, Germany; ^11^ Department of Psychiatry and Psychotherapy, Vitos Klinikum Riedstadt, Riedstadt, Germany; ^12^ Department of Psychiatry and Psychotherapy, Imland Klinik Rendsburg, Rendsburg, Germany; ^13^ Department of Psychiatry and Psychotherapy and Psychosomatic Medicine, Carl-von-Basedow-Klinikum Saalekreis, Querfurt, Germany

**Keywords:** flexible and integrative psychiatric treatment models, implementation, global treatment budget, mental health funding, personal services, cross-sectoral

## Abstract

**Background:**

Despite evidence from other countries for its effectiveness, flexible and integrative psychiatric treatment (FIT) is not part of the German standard healthcare system. Since 2013, German legislative reform has enabled a test implementation of FIT based on a global treatment budget. Because the budget is not restricted to any particular activity, this legislation opens the possibility of enhancing linkages between inpatient-, outpatient- and day-patient treatment structures. As staff involvement is a relevant component in successful implementation, we aimed in this study to judge the degree of FIT implementation based on staff members’ experiences and evaluations of FIT.

**Method:**

Within an exploratory study design, we administered a standardized written survey to rate experiences and evaluations of physicians, psychologists, and nurses in the first 13 FIT projects between October 2016 and February 2017. The sample consisted of 352 nurses, 127 physicians, 84 psychologists, and 132 special therapists. We identified critical factors for successful implementation from the staff perspective by logistic regression analysis.

**Results:**

Staff evaluations of the degree of FIT implementation were generally favorable, although some staff reported no experiences with one or several FIT-specific components. We found considerable differences in the assessments between the occupational groups. The only common factor for successful FIT implementation shared by physicians, psychologists, and nurses was the opportunity to join training programs on the objectives of FIT. Other critical factors for successful implementation were work conditions, the number of nurses/special therapists per physician/psychologist, and project duration. These factors together explained 49% of the variance of physician/psychologist evaluations and 34% for nurse evaluations. Individual staff members’ characteristics were less important than structural- or FIT characteristics as explanatory factors for the degree of FIT implementation.

**Implications:**

Results point to the importance of new forms of multi-professional cooperation, training programs, improvement of work conditions, and guidance of the implementation process by systematic Change Management for future implementations of FIT. Our exploratory findings require further validation to guide practical improvements in FIT implementation. Longitudinal observations and a multilevel analysis should yield a better understanding of the relevant variables from different organization levels and their possible interactions.

## Introduction

Despite good evidence for its effectiveness, internationally well-established community-based flexible and integrative psychiatric treatment (henceforth abbreviated as FIT) is not implemented in the standard German healthcare system (1–5). Instead, inpatient treatment based on per diem and performance-oriented payment approaches remains the major healthcare sector in Germany. This state of affairs may not entirely satisfy the requirements of needs-oriented and patient-centered care and may lead to over- or under-utilization of healthcare services, or to other forms of misdirected use (6–10).

Since the year 2003, only single psychiatric departments in Germany have negotiated individual contracts for FIT with health insurance companies based on a “regional budget”, otherwise known variously as “capitation model”, “capitated payment system”, “mental health capitation model”, or “capitated model for psychiatric care”. A nationwide implementation of FIT was enabled for the first time in Germany by a legislative reform in 2013 (§ 64b German Social Code Book V). This law allows for the test implementation of FIT in the special case of the treatment of patients suffering from psychiatric conditions (11). Scientific evaluations of the initial FIT projects have been encouraging for the further development of FIT projects (12–14).

FIT projects are based on a global treatment budget (henceforth abbreviated as GTB), which is an annually allocated and project-based fixed budget to cover all forms of treatment for a defined patient population. The GTB can be described as occupying a middle ground between block contracts and capitation payments. Block contracts have financing based on a fixed lump sum, which is roughly determined by precedents such as the historical expenditures for a particular service, but can be adjusted according to patient needs (15). The lump sum is set irrespective of the number of patients treated or the amount of therapeutic engagement that is undertaken. Capitation payment involves payment of an annual lump sum for a given number of patients in the target population, irrespective of how many services the patients may receive (16–19). While capitation payment entails uniform remuneration per treated patient (bottom-up computation), GTB is based upon case numbers of the years prior to the contract (top-down computation). In practice, an initial normative or empirical calculation of remuneration per capita is multiplied by the number of such patients treated in the fiscal year. In its original conception, a bundled or rather episodic payment approach serves for FIT financing. Under this approach, a single annual payment is made for a package of services, which is calculated from the expected costs for the clinically defined care episodes (20).

An important aim of FIT is to redirect the focus of health care on individual patient needs and regional requirements, thus diverging from traditional provider-driven and mainly inpatient treatment structures (17, 21, 22). Because its budget is not restricted to any particular activity undertaken, the advent of FIT should foster cross-sectoral care by enhancing linkages among outpatient-, inpatient-, and day-patient treatment structures. Based on outcome research such as that reported in the present study, the German government shall decide by 2024 if FIT should become a standard part of the national healthcare system.

Initial research results, consisting of data from clinical account databases, cost analysis, patient- and individual staff-related findings are already available for the first FIT projects tested in Germany (21, 23, 24). However, available findings do not suffice to measure the degree of implementation for FIT with due consideration of their character as personal services. For such services, the outcome quality resides primarily in the quality of interaction between the involved parties. In the case of clinical psychiatry, outcome quality reflects the interactions between patients and the treatment staff, who implicitly and explicitly communicate their attitudes towards (changed) work specifications (25–27). Barriers or conversely facilitators for FIT implementation may arise at various levels of healthcare delivery (28). In the case of personal services, it is the staff attitude towards structural and organizational changes that constitutes a critical factor for successful implementation (29–34). A key requirement for successful implementation is the extent to which staff are informed in advance of FIT-related structural and procedural changes, and are kept up to data about the experience gained upon adopting new measures in the occupational routine. However, merely experiencing these changes is not sufficient for successful implementation, which substantially depends on the care providers’ evaluation of the modifications, including an integration into professional attitudes and daily work procedures. Ideally, health care providers should consider themselves as agents of change, rather than as passive recipients of evolving workplace specifications (25, 26, 29, 32, 33, 35). Failure of implementation often occurs when there is tacit opposition before even starting the process of change, resulting in an inability of the organization to “unfreeze” and adopt a stance of readiness for change (29, 33). Therefore, staff involvement is a highly relevant factor in evaluating the processes that lead to successful implementation.

This paper is part of the multi-center and mixed-methods exploratory study ‘EvaMod64b’, which aims to describe the multifaceted effects of the first Germany-wide FIT projects on patients, informal caregivers, and staff in relation to the degree of implementation of FIT projects (23, 24, 36). We now report results of our standardized written survey of evaluations by physicians, psychologists, and nurses on their experience with initiating FIT-related structures and procedures in the setting of psychiatric departments across Germany. We posed the following five questions to assess the degree of FIT implementation from the staff perspective: (1) To what extent are staff informed and experienced with FIT-related structures and procedures? (2) Does the degree of staff experience with FIT relate to the project duration? (3) How are characteristics of FIT evaluated by staff? (4) Which individual, organizational, and structural characteristics correspond best with the staff evaluations? (5) What are the critical factors for successful FIT implementation from the staff perspective?

## Materials and Methods

### Setting and Sampling

The study was approved by the Ethics Committee Brandenburg [2016, No. S 7 (a)], thus adhering to the ethical standards laid down in the 1964 Declaration of Helsinki and its later amendments. The staff survey was approved by the respective institutional work councils. Potential participants received a verbal project-description, were informed about the voluntary nature of their participation, and were guaranteed anonymity.

In 2015, all 11 FIT projects established in 15 psychiatry departments in nine different German cities and regions were invited to join the study. Among these, leaders of nine projects from 13 departments agreed to participate (ten adult psychiatry and three child and adolescent psychiatry departments). Of the 13 departments, one withdrew from the study for organizational reasons. We inquired about sociodemographic, professional, and structural characteristics of the workplaces (as illustrated in point 2.2). The start dates of FIT extended from January 2013 to January 2016. Eight departments had established FIT for more than two years and the remaining four departments for two years or less at the time of data collection. Seven departments had a history of FIT in the form of individually negotiated contracts with health insurance companies, which were either according to GTB regulations or those of integrated care programs. The examined departments were either public (seven departments) or non-profit (five departments), providing care for regional populations ranging from 85,000 to 425,000 people. Eight departments were under contract with all national insurance companies. In the four departments having contracts with only one or two insurers, not all patients received FIT.

We administered the standardized written survey (as illustrated in point 2.2) of physicians, psychologists, nurses, and special therapists (e.g. occupational therapists, physiotherapists and music therapists) between October 2016 and February 2017. Only staff working in settings with partial or complete FIT implementation were interviewed. The sample consisted of 352 nurses, 127 physicians, 84 psychologists, and 132 special therapists (**Table 1**). Because of the considerable heterogeneity of special therapists’ professional backgrounds and fields of activities, we confined our analysis to data provided by physicians, psychologists, and nurses. The participants were of mean age of 41 years and had on average 12 years of work experience in psychiatry. The majority of participants was female (73%) and worked full time (62%) in general psychiatry (40%). While physicians (75%) and psychologists (61%) mainly worked in the outpatient setting, nurses (77%) mainly worked in the inpatient treatment setting. The mean response rates by institution ranged between 31-88% for physicians/psychologists and 14-87% for nurses.

**Table 1:** Sociodemographic and professional characteristics of staff.

**Characteristic**	**Physicians (*n* = 127)**	**Psychologists (*n* = 84)**	**Nurses (*n* = 352)**
**Age and Gender^A^**
Age (years, ± SD)	42.5 ( *±* 10.6) (*N* = 123)	35.9 (*±* 11.5) (*N* = 83)	43.3 ( *±* 11.9) (*N* = 290)
Female	52.8%* (*N* = 67)	92.9%* (*N* = 78)	72.6% (*N* = 228)
Male	44.9%* (*N* = 57)	6%* (*N* = 5)	27.4% (*N* = 86)
**Experience^A^**			
Work experience in psychiatry (years) ( ± SD)	11.5 (*±* 9.6) (*N* = 122)	7.3 (*±* 8.2) (*N* = 82)	15.9 (*±* 9.9) (*N* = 287)
Length of employment in current institution (years) ( ± SD)	7.5 (*±* 6.9) (*N* = 113)	5.3 (*±* 6.3) (*N* = 76)	14.2 (*±* 9.3) (*N* = 273)
**Working hours^A^**			
Serving full-time (100%)	78.7%* (*N* = 100)	39.3% (*N* = 33)	67%* (*N* = 217)
Serving part-time (< 100%)	19.7%* (*N* = 25)	60.7% (*N* = 51)	33%* (*N* = 107)
**Position^A^**			
Assistant physician	48.9% (*N* = 62)	n/a	n/a
Medical specialist without leading position	15.7% (*N* = 20)	n/a	n/a
Senior physician	30.7% (*n* = 39)	n/a	n/a
Chief physician	4.7% (*n* = 6)	n/a	n/a
Psychologists	n/a	94% (*n* = 79)	n/a
Leading psychologist	n/a	6% (*n* = 5)	n/a
Supervising nurse	n/a	n/a	18.2% (*n* = 59)
Nurse without leading position	n/a	n/a	81.8% (*n* = 266)
**Education of nurses^A^** (several answers possible)			
Nurse (3 years trained)	n/a	n/a	84.3% (*n* = 296)
Nursing assistant (1 year trained)	n/a	n/a	0.6% (*n* = 2)
Degree (Bachelor, Master)	n/a	n/a	2.8% (*n* = 10)
Specially trained psychiatric nurse (3 years trained + 2 years special training)	n/a	n/a	15.3% (*n* = 54)
**Treatment setting^A^** (several answers possible)			
Inpatient treatment setting	64.6% (*n* = 82)	46.4% (*n* = 39)	76.7% (*n* = 270)
Part-time inpatient setting	52% (*n* = 66)	57.1% (*n* = 48)	29.8% (*n* = 105)
Outpatient	74.8% (*n* = 95)	60.7% (*n* = 51)	30.1% (*n* = 106)
Others	11.8% (*n* = 15)	9.5% (*n* = 8)	1.7% (*n* = 6)
**Current field of activity^A^**			
General psychiatry	51.2% (*n* = 65)	31%* (*n* = 26)	37%* (*n* = 120)
Addiction medicine	10.2% (*n* = 13)	8.3%* (*n* = 7)	12.3%* (*n* = 40)
Psychosomatic medicine	7.1% (*n* = 9)	21.4%* (*n* = 18)	13.4%* (*n* = 43)
Gerontological psychiatry	3.9% (*n* = 5)	9.5%* (*n* = 8)	6.5%* (*n* = 21)
Child and adolescent psychiatry	12.6% (*n* = 16)	10.7%* (*n* = 9)	15.4%* (*n* = 50)
Mixed fields and others	15% (*n* = 19)	17.8%* (*n* = 15)	15.4%* (*n* = 50)

^A^Reference category; n/a = not applicable; * difference to 100% = missing values; SD = standard deviation.

### Measuring Staff Experiences and Evaluations of FIT-Specific Components and Work Conditions

We administered a questionnaire consisting of three parts: (1) sociodemographic, professional, and structural characteristics of staff and workplaces (29 items for physicians/psychologists, 34 items for nurses), (2) specific components of FIT (28 items), (3) work conditions (28 items for physicians/psychologists, 32 items for nurses). Part 1 inquired about sociodemographic factors such as age and gender, along with professional characteristics. These included noting if staff were serving full-time versus part-time, vocational training, years of professional engagement in psychiatry, and current position. Part 1 also covered structural aspects of the workplaces such as the treatment setting and number of colleagues. The questionnaire, which consisted of 94 items for nurses and 85 for physicians/psychologists, was administered in a pencil and paper format requiring 15-20 minutes for completion. Other core elements of our study encompassed by parts 2 and 3 are presented in more detail below.

#### FIT-Specific Components

To operationalize the staff perspective as a measure of the degree of FIT implementation, we defined two statistical metrics. These were based on the distinction between staff members’ experiences and evaluations of FIT-related structures and procedures. The first of these metrics, ‘experiences’ (henceforth abbreviated as EX), is an index of whether staff members were informed about FIT-related structural and procedural changes and to what extent they experienced these changes in their occupational routine. The second metric, ‘evaluations’ (henceforth abbreviated as EV), is an index of attitudes towards and identification with the changes that were experienced.

The FIT projects differed with respect to factors such as project duration, size of catchment area, urban/rural area, treatment structures, and procedures. To accommodate this heterogeneity, we defined a set of 11 operationalized FIT-specific components in a pilot stage of ‘EvaMod64b’, while following the Grounded Theory Methodology (23, 24, 36). After defining these components, we developed a 28-item questionnaire (‘Characteristics, Structures and Procedures of Model Projects’), which operationalized these components to measure EX and EV (**Table 2**). The specific component ‘accessibility of services’, meaning the geographical and team accessibility, was not included in the questionnaire because this component related only to patients. We integrated two additional items, both referring to ‘attitude change’, which had not been defined in the initial component set, but emerged at a later stage of the study ‘EvaMod64b’.

**Table 2:** Definition, main and subordinate categories of FIT-specific components for the questionnaire ‘Characteristics, Structures and Procedures of Model Projects’.

FIT-specific component and definition of component	Main and subordinate categories in questionnaire
**Shifting in- to outpatient setting** Shift of treatment from inpatient- towards day-patient and/or outpatient treatment setting	*Shifting of treatment units from inpatient- towards day-patient- and/or outpatient treatment setting
	Systematic range of day-patient treatment
**Flexible care management across settings** Unproblematic shift of treatment setting (outpatient, day-patient, inpatient) (prompt, little bureaucracy)	*Flexible transition from one to another setting
	Shifting wards to treatment focuses
**Continuity of treatment team** Implementation of team- and individual-related continuity	*Continuity of treatment team across settings
	Continuity of treatment across day-patient and inpatient treatment
**Multi-professional cooperation** Intense multi-professional cooperation	*Systematic multi-professional cooperation
	Obligatory multi-professional meetings
	Networking of visiting outpatient service and inpatient treatment team
**Therapeutic group sessions across all settings** Therapeutic groups with members from all treatment settings (outpatient, day-patient, inpatient)	*Therapeutic group sessions across all settings
	Development of patient- and staff groups across wards/functional areas
	Networks for patients and integration in groups across all settings
**Outreach home care** Multi-professional treatment at home ≥ 1 week	*Systematic outreach home care offer (multi-professional, visiting, ≥ 1 week)
	Systematic offer for home visits Intensification of cooperation with residential homes
**Involvement of informal caregivers** Informal caregivers as therapeutic tool	*Systematic involvement of informal caregivers
**Accessibility of services** Geographical accessibility and accessibility of teams	Inapplicable for staff, relevant only for patients
**Sovereign steering of services** Freedom of therapeutic decisions	*Greater scope of action (e.g. leave of absence for patients; weekend holiday)
	Individualized therapy plans take the place of standardized rules
	Reduction of end of treatments through more possibility of differentiation, offers and compromises
	Flexibility of treatment procedure [e.g. certain treatment offers without prior approval of German medical service of healthcare insurance companies (MDK)] with larger margins for patients
**Cooperation across sectors** Cooperation with ambulant care systems	*Management of treatment across sectors
	Quality circles across treatment sectors
	Development of networking groups with independent sponsors
**Expansion of professional expertise** Professionalization of staff	*Increase of independent work
	Specific training programs to the objectives of the model projects
	Dissolving boarders between professions and teamwork is getting more important
**^†^Attitude change** Change of attitude due to implementation of FIT	Intensive patient involvement in therapy through informed consent
	Closeness to the daily routine of patients and informal caregivers plays a key role in the treatment

*Main category for one FIT-specific component; ^†^Additional category originated from a later stage of the study, not belonging to the initial FIT-specific components.

We posed the following key question to quantify EX and EV of FIT-specific components from the staff perspective, each according to a one-answer scale with two subsections: “How do you rate the impact of structures/procedures for the treatment/care for patients with mental illness in your hospital such as are already partially realized/enabled by FIT on the outcomes of your occupational routine in the past months?” In the first part, permitted responses about EX were “nonexistent” and “present, but not yet assessable”. In the second part, permitted staff members responses for each item about EV were “present and assessable and my opinion of it is (…)” “very positive”, “rather positive”, “partly”, “rather negative”, and “very negative”.

#### Work Conditions

Participants were asked to rate their present work conditions regarding supervision and hierarchy, conflict resolution ability of the team, work conditions on the ward/functional area, cooperation among occupational groups, requirements of patients, and opportunities for making joint decisions. Therefore, we adopted 23 questions for physicians/psychologists and 27 for nurses and special therapists from the German ‘Questionnaire on Work Situation for Doctors’ (FAÄ) (37) and the German ‘Questionnaire for Nurses in Psychiatry’ (FAPP) (38, 39), as well as five questions from the study “Registered Nurses Forecast” (RN4CAST) (40). We modified the 6-point scale of the FAÄ and the FAPP, which was initially scored as 3 (“rather good”) -2-1-1-2-3 (“rather poor”) (2-1-1-2 were not precisely defined in the original version) to a 1-2-3-4-5-6 scale of descending quality. Here, scores ranged from 1, defined as “very good” or some comparable statement such as “very often”, to 6, which was “very poor” or a comparable statement such as “occasionally”.

The comprehensiveness of the questionnaire was reviewed by project members trained in empirical social research and with prior experience within the field of FIT, and by physicians, psychologists, nurses, and other professionals from every hierarchical level of four FIT departments. Subsequently, for the 28 item questionnaire (‘Characteristics, Structures and Procedures of Model Projects’), each specific component was defined by one main and one or more subordinate categories (**Table 2**).

### Data Analysis

The data analysis of EX and EV covered the ten main categories of the 28-item questionnaire ‘Characteristics, Structures and Procedures of Model Projects’. The two items noted above referring to ‘attitude change’ were excluded from the analysis because they were not among the initial FIT-specific components. In addition, the item ‘specific training programs to the objective of the model project’ (henceforth abbreviated as ‘training programs’), initially assigned to a subordinate category of the specific component ‘expansion of professional expertise’ (**Table 2**), was integrated into the analysis, as noted below.

Individual staff members’ ratings of organizational and structural characteristics of FIT departments as well as EX and EV were assessed *via* descriptive statistics. Participating departments were compared using structural data such as project duration and history of FIT in the form of individually negotiated contracts with insurance companies, extent of cooperation with health insurance companies, departments’ sponsorship, and catchment size. Categorical data were tested using the χ²- test or Fisher’s exact test in case of small cell counts.

EX was calculated descriptively *via* the three responses 1 = ‘‘nonexistent”, 2 = “present, but not yet assessable”, and 3 = “present and assessable”. To calculate the relation between EX and the dichotomized variable ‘project duration’ (dichotomized as short = ≤ 2 years versus long = > 2 years), a Chi-square test was performed.

EV was calculated only in the event that EX was rated to be “present and assessable”. EV scores then ranged from 1 (low/negative evaluation of implementation) to a maximum of 5 (high/positive evaluation).

The correlations between EV and the variables ‘project duration’ (dichotomized as above), ‘training programs’ (dichotomized as “rather positive”, “very positive” versus “very negative”, “negative”, “partly”), as well as individual staff members’ judgement of organizational and structural characteristics of FIT departments, were analyzed *via* Spearman correlation.

The five research questions posed under point 1 were tested in an exploratory manner with α of 5% with no use of alpha-adaption. Test results with *p* < α (5%) were here deemed significant.

For the binary logistic regression, EV was dichotomized to 1 = “very negative”, “negative”, “partly”, and 2 = “rather positive”, “very positive”. For physicians/psychologists, logistic regression was performed with EV as the dependent variable and the independent variables ‘age’ (> versus ≤ mean), ‘duration of employment in psychiatry’ (> versus ≤ mean), ‘number of nurses/special therapists per physician/psychologist’ (> versus ≤ median 3.3), ‘project duration’ (dichotomized as above), ‘training programs’ (dichotomized as above), and ‘sum of positively rated work conditions’ (≥ versus < 50% of work conditions positively rated). We defined the number of nurses/special therapists per physician/psychologist as the number of nurses/special therapists per primary physician or psychologist, all considered as one group. For nurses, the regression was calculated with EV as the dependent variable and the independent variables ‘training programs’ (dichotomized as above), ‘sum of positively rated work conditions’ (≥ versus < 50% of work conditions positively rated), ‘project duration’ (dichotomized as above), and ‘supervisor for other nurses’ (being supervisor for other nurses versus no status as supervisor). For both groups, we made the binary logistic regression based on the results of the exploratory Spearman correlation. Statistical results were computed by SPSS 15 and 22.

## Results

For the questionnaire ‘Characteristics, Structures and Procedures of Model Projects’, Cronbach’s α for physicians’/psychologists’ questions was 0.86 for EX and 0.88 for EV, which are both regarded as good (= > 0.8) according to the definition of Cronbach (41). For nurses’ questions, Cronbach’s α was excellent (= > 0.9), with 0.99 for EX and 0.92 for EV (41). After modification of the 6-point scale of the ‘Questionnaire on Work Situation for Doctors’ (FAÄ) and the corresponding questionnaire for nurses (FAPP), as mentioned in point 2.2, Cronbach’s α remained good (> 0.8) for physicians’/psychologists’ questions and likewise for nurses (0.84 for EX and 0.88 for EV).

In the following sections, we present EX, EV, and critical factors for success in the evaluations of FIT from the staff perspective.

### Experiences With FIT-Specific Components (EX)

**Figure 1:**
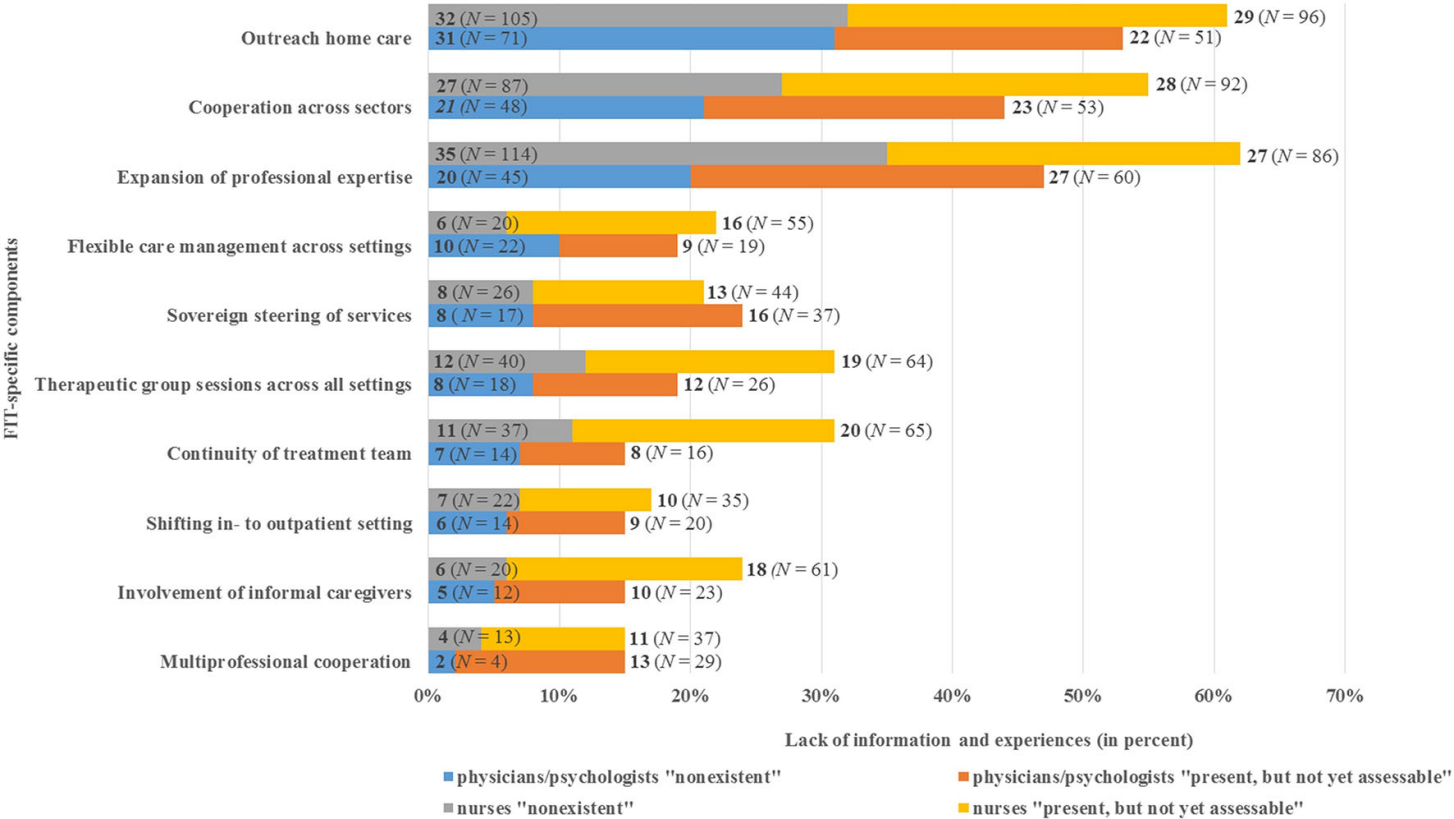
Physicians’/psychologists’ (*N* = 211) and nurses’ (*N* = 352) lack of information and experience with FIT-specific components (EX).

EX was higher for nurses compared to physicians/psychologists with respect to eight of the ten FIT-specific components, indicating that nurses were less informed and experienced with FIT-specific components at the time of data collection (**Figure 1**). Comparing the answers by physicians/psychologists with those of nurses, the largest difference related to the component ‘expansion of professional expertise’ (20% of physicians/psychologists vs. 35% of nurses stated “nonexistent”). Remarkably, 36% of physicians/psychologists and 27% of nurses stated that no training programs on objectives of FIT existed in their departments. Additionally, 22% of the physicians/psychologists and 28% of the nurses stated that training programs were present, but were not assessable to them.

As shown by the EX results, up to 31% of physicians/psychologists and 35% of nurses were not experienced with at least one of the FIT-specific components (**Figure 1**). For instance, 31% of physicians/psychologists and 32% of nurses reported no experience with the component ‘outreach home care’. In addition, 21% of physicians/psychologists and 27% of nurses stated that their department did not cooperate with other institutions across various health service sectors. The maximum values of EX were found for the component ‘multi-professional cooperation’, with 2% of physicians/psychologists and 4% of nurses assessing this component as “nonexistent”.

While for nurses there was no significant relation between EX and the project duration (*x* ^2^ (2) = 3.323, p = 0.190, *n* = 304), the Chi-square test was significant for physicians/psychologists (*x* ^2^(2) = 9.948, *p* = 0.007, Cramer’s *V* = 0.235, *n* = 180) (42). This indicates that nurses had less experience than did physicians/psychologists with FIT-specific components, even after two years of project duration.

### Evaluations of FIT-Specific Components (EV)

**Figure 2:**
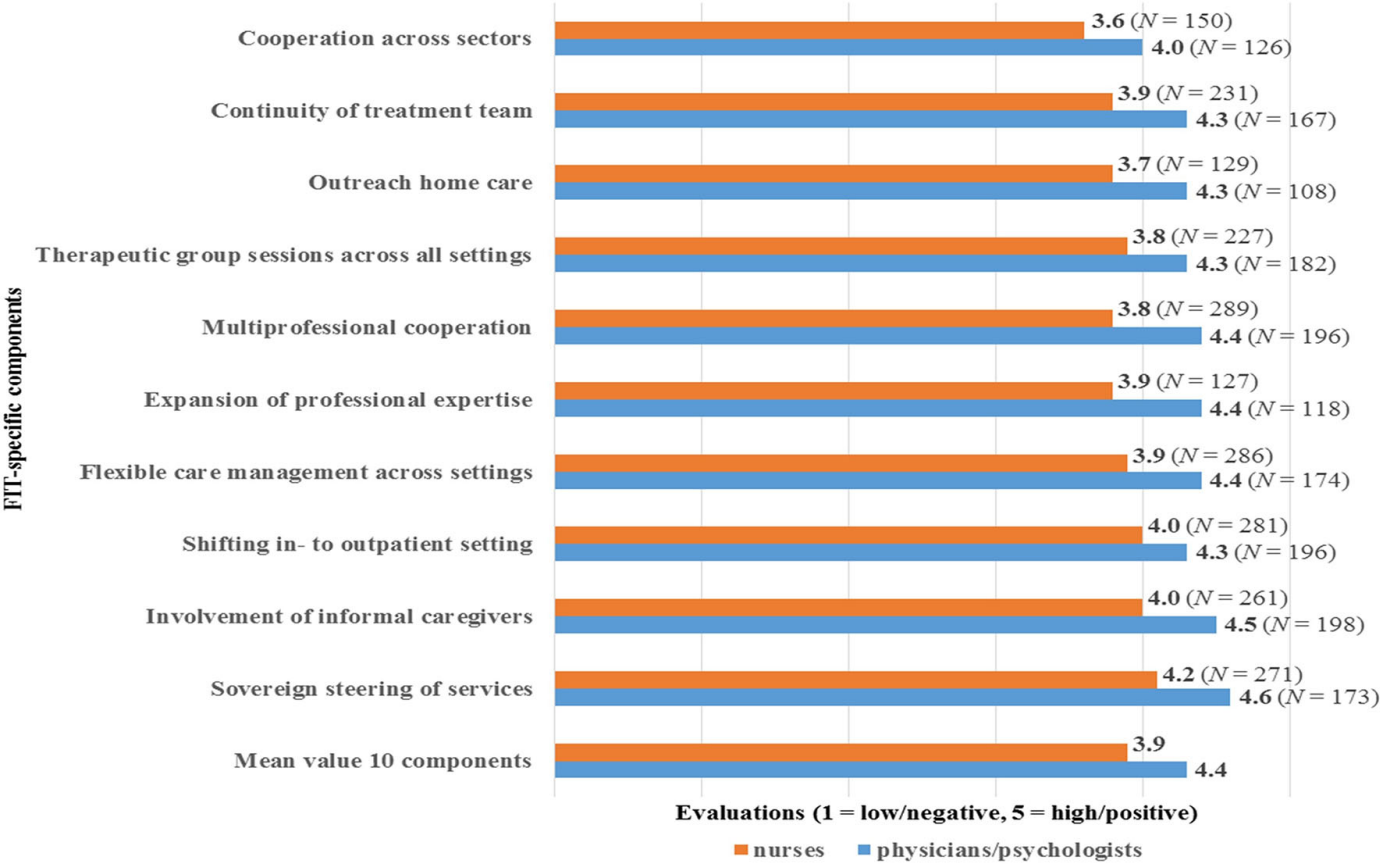
Evaluations of FIT-components (EV) by physicians/psychologists (*N* = 211) and nurses (*N* = 352).

The mean value for EV, covering all ten FIT-specific components, was 4.4 of a maximum of 5 for physicians/psychologists and 3.9/5 for nurses (**Figure 2**). These values indicate rather positive evaluations of FIT by the surveyed nurses and to an even greater extent by physicians/psychologists.

The highest mean values of EV were found for the component ‘sovereign steering of services’ (4.6 for physicians/psychologists, 4.2 for nurses), and the lowest values for the component ‘cooperation across sectors’ (4.0 for physicians/psychologists, 3.6 for nurses). Overall, nurses’ EV scores were at least 0.3 points lower compared to these of physicians/psychologists. The comparison showed maximum differences between the EV of the occupational groups regarding the components ‘outreach home care’ and ‘multi-professional cooperation’ (both with a difference of 0.6). The least difference between physicians/psychologists and nurses occurred in relation to the component ‘shifting in- to outpatient setting’ with a difference of 0.3.

Bivariate analysis of the EV and individual, organizational, and structural characteristics for physicians/psychologists showed significant positive correlations between EV and higher age (*p* = 0.017), longer duration of employment in psychiatry (*p =* 0.015), and the higher number of nurses/special therapists per physician/psychologist (*p* = 0.006) (**Table 3**). For nurses, bivariate analysis showed a significant negative correlation between EV and the variable ‘supervisor for other nurses’ (*p* = 0.022). For both occupational groups, a positively rated opportunity to join training programs (both groups *p* < 0.001), a higher sum of positively rated work conditions (physicians/psychologists *p* = 0.006, nurses *p* < 0.001), and longer project duration (physicians/psychologists *p* = 0.012, nurses *p* = 0.016) correlated significantly with a higher value of EV.

**Table 3:** Results of the bivariate Spearman analysis for individual, organizational, structural characteristics, and the evaluations (EV) of physicians/psychologists and nurses.

Characteristics	Physicians/psychologists (*n* = 211)	Nurses (*n* = 352)
**Individual characteristics^A^**		
Age (> versus ≤ mean)	*r* = 0.17* (*p* = 0.017)	*r* = 0.07 (*p* = 0.29)
Gender (male versus female)	*r* = -0.12 (*p* = 0.10)	*r* = 0.06 (*p* = 0.32)
Qualification (not certified versus certified)	n/a	*r* = -0.04 (*p* = 0.48)
Training duration (other versus 1-year training (nurses)	n/a	*r* = -0.03 (*p* = 0.62)
Professional status as a nurse: being supervisor for other nurses versus no status as supervisor	n/a	*r* = -0.13* (*p* = 0.022)
Professional status as physician (assistant physician versus specialist without leadership position, senior physician, chief physician)	*r* = -0.00 (*p* = 0.96)	n/a
Duration of employment in psychiatry (> versus ≤ mean)	*r* = 0.17* (*p* = 0.015)	*r* = 0.03 (*p* = 0.58)
Duration of employment in current department (> versus ≤ mean)	*r* = 0.13 (*p* = 0.07)	*r* = 0.01 (*p* = 0.88)
Full-time (100%) versus part-time (< 100%) employment	*r* = -0.05 (*p* = 0.45)	*r* = -0.03 (*p* = 0.67)
**Organizational characteristics^A^**		
Number of nurses/special therapists per physician/psychologist (> versus ≤ median 3.3)	*r* = 0.18** (*p* = 0.006)	n/a
Existence versus non-existence of a FIT-feedback system	*r* = -0.03 (*p* = 0.66)	*r* = 0.02 (*p* = 0.78)
Opportunity to join specific training programs to the objective of the model project (“rather positive,” “very positive” versus “very negative,” “negative,” “partly”)	*r* = 0.37*** (*p* < 0.001)	*r* = 0.04*** *(p* < 0.001)
Sum of positively rated work conditions from 23 (physicians/psychologists) or 27 (nurses) items (≥ versus < 50% of work conditions positively rated)	*r* = 0.19** (*p* = 0.006)	*r* = 0.38*** (*p* < 0.001)
**Structural characteristics^A^**		
Department’s sponsorship (public versus non-profit, private)	*r* = 0.00 (*p* = 0.97)	*r* = 0.08 (*p* = 0.16)
Project duration (> versus ≤ 2 years)	*r* = 0.17* (*p* = 0.012)	*r* = 0.13* (*p* = 0.016)
Competitive versus no competitive situation to another hospital	*r* = 0.12 (*p* = 0.11)	*r* = 0.02 (*p* = 0.75)

^A^ Reference category; n/a, not applicable; *p < .05, **p < .01, ***p < .001; r = Spearman Rank correlation coefficient.

### Critical Factors for Success

The model for physicians/psychologists, as introduced in point 2.3, was significant for EV (*x* ^2^(6) = 24.477, *p* < 0.001, *n* = 68), but not for every coefficient within the above exploratory bivariate analysis (**Table 4**). The chance for a positive evaluation (EV) of the FIT-specific components for physicians/psychologists increased 16.5-fold when the item ‘training programs’ was positively evaluated (*p* = 0.008), 13.2-fold for a higher number of nurses/special therapists per physician/psychologist (*p* = 0.013), and 10.4-fold for a project duration exceeding two years (*p =* 0.036). Inclusion of the coefficients ‘age’, ‘sum of positively rated work conditions’, and ‘duration of employment in psychiatry’ did not contribute significantly to the prediction of the EV outcome. While showing no significant effect in the regression analysis, the coefficient ‘age’ was negatively associated with EV. Thus, 49% of the variance of EV could be explained by only three significant independent variables, corresponding to a strong effect according to Cohen (42).

**Table 4:** Full binary logistic regression for evaluations (EV) of physicians/psychologists and selected independent variables (*N* = 68).

Variable	*b (SE)*	*p*	95% CI for Odds Ratio		
			Lower Bound	Odds Ratio	Upper Bound
Duration of employment in psychiatry (> versus ≤ mean)	0.19 (0.09)	0.053	0.99	1.21	1.46
Number of nurses/special therapists per physician/psychologist (> versus ≤ median 3.3)	2.57 (1.03)	0.013*	1.72	13.18	100.97
Project duration (> versus ≤ 2 years)	2.34 (1.12)	0.036*	1.15	10.41	93.58
Opportunity to join specific training programs to the objective of the model project (“rather positive”, “very positive” versus “very negative”, “negative”, “partly”)	2.80 (1.06)	0.008**	2.06	16.49	131.77
Sum of positively rated work conditions from 23 (physicians/psychologists) (≥ versus < 50% of work conditions positively rated)	0.74 (0.83)	0.376	0.40	2.09	10.77
Age (> versus ≤ mean)	-0.10 (0.06)	0.106	0.78	0.89	1.02

*p < .05, **p < .01; CI, confidence interval; b, regression coefficient; SE, standard error of regression coefficient.

The model for nurses was significant for EV (*x* ^2^(4) = 32.605, *p* < 0.001, *n* = 112), but not for each coefficient selected on the basis of the exploratory bivariate analysis described above (**Table 5**). For nurses, the chance for a positive evaluation (EV) of the FIT-specific components increased 5.1-fold when a higher sum of work conditions was rated positively (*p* = 0.001) and 4.9-fold when ‘training programs’ was positively evaluated (*p* < 0.001). Inclusion of the coefficients ‘supervisor for other nurses’ and ‘project duration’ did not contribute significantly to the prediction of the EV outcome, even though both coefficients showed a negative association with EV. Thus, 34% of the variance of EV could be explained by the three significant independent variables, corresponding to a strong effect according to Cohen (42).

**Table 5:** Full binary logistic regression for evaluations (EV) of nurses and selected independent variables (*N* = 112).

Variable	*b (SE)*	*p*	95% CI for Odds Ratio		
			Lower Bound	Odds Ratio	Upper Bound
Project duration (> versus ≤ 2 years)	-0.16 (0.46)	0.731	0.34	0.85	2.12
Professional status as a nurse: being supervisor for other nurses versus no status as supervisor	-0.72 (0.53)	0.176	0.16	0.48	1.38
Opportunity to join specific training programs to the objective of the model project (“rather positive”, “very positive” versus “very negative”, “negative”, “partly”)	1.59 (0.45)	< 0.001***	2.03	4.90	11.83
Sum of positively rated work conditions from 27 (nurses) (≥ versus < 50% of work conditions positively rated)	1.63 (0.47)	0.001**	2.03	5.12	12.91

**p < .01, ***p < .001; CI, confidence interval; b, regression coefficient; SE, standard error of regression coefficient.

## Discussion

### Degree of FIT Implementation

Overall, the experiences of FIT were evaluated rather positively by nurses and even more so by physicians and psychologists. Implementation, measured by scores in staff evaluations, was generally most advanced in the FIT-specific component ‘sovereign steering of services’ and least successfully in ‘cooperation across sectors’. The importance of both of these aspects is a familiar result from other hospital workplace research. Autonomy in clinical decision processes is considered one of the most important components of work satisfaction (43–45). In contrast, a perceived lack of autonomy may contribute to work dissatisfaction, higher rates of staff turnover, lower effectiveness in clinical settings, and higher healthcare costs (45, 46). The occurrence of inadequate cooperation across sectors is a well-known deficiency of the German healthcare system. The need to correct this lack of cooperation was a key motivation for the legislative reform allowing FIT implementation based on a GTB (11, 23, 47).

While we registered a generally favorable assessment of the degree of FIT implementation according to staff evaluations, a significant proportion of the staff nonetheless reported having had no experience with one or more FIT-specific components. ‘Outreach home care’ and ‘cooperation across sectors’ were deemed the least advanced of the implemented components according to staff experiences. We note that several departments had not implemented the component of ‘outreach home care’ at the time of data collection. As suggested by the scant experience and relatively poor evaluations of ‘cooperation across sectors’, this item emerges as an FIT-specific component particularly in need of efforts for improved implementation.

Regarding the interpretation of EV, we note the importance of considering that changes in the workplace associated with FIT (for example, the delegation of more responsibility, demands for professional development, and greater inter-professional cooperation) were not to the liking of every employee. We suppose that some employees were averse to, or felt overburdened by these changes in routine.

As discussed in the section below, present results indicate that individual characteristics of staff (e.g. age, qualification) played a less important role concerning the degree of implementation than did characteristics of FIT (e.g. project duration) and structural aspects of FIT departments (e.g. the sum of positively rated work conditions).

### Factors for Success in FIT Implementation

Regression analysis identified four factors for success, namely (1) positive evaluation of the opportunity to join training programs about the objectives of FIT, (2) project duration, (3) work conditions, and (4) the number of nurses/special therapists per physician/psychologist. The only factor for success in FIT implementation from the perspectives both of physicians/psychologists and of nurses was the positive evaluation of the opportunity to join training programs about the objectives of FIT. The chance for a positive evaluation of FIT was increased 16.5-fold for physicians/psychologists and 4.9-fold for nurses by this variable. Given its reported importance for staff, it seems remarkable that 36% of physicians/psychologists and 27% of nurses stated that no training programs existed in their departments. This discrepancy emphasizes that the return on investment for training programs should not be underestimated as a factor for better understanding, participation, and integration of planned modifications, thus positively influencing attitudes and procedures.

For physicians/psychologists, the second most critical factor for successful FIT implementation was a higher number of nurses/special therapists per physician/psychologist. We suppose that higher staffing with nurses/special therapists may relieve some organizational or other burden placed on physicians/psychologists, especially during the early phase of FIT implementation, when our prior research indicates an increased workload (23).

Project duration was the third critical factor for success from the perspective of physicians/psychologists. This finding illustrates that clinical staff need sufficient time to grow accustomed to the FIT-related changes and to undergo certain modifications of professional attitudes and daily work procedures. Especially during the departure phase, that is to say the first two years of FIT implementation, staff has to accommodate a drastic reduction of the number of beds and the adoption of new treatment concepts (23). In this early phase, it was sometimes necessary for staff to manage double routines, especially in those departments not under contract with all national health insurance companies (23). The finding that project duration is important for the degree of FIT implementation is also consistent with earlier results showing that a longer duration of Crisis Resolution Team, Assertive Outreach Team, or Community Mental Health Treatment predicted for fewer experiences of emotional exhaustion and depersonalization in response to procedural changes (48).

From the perspective of nurses, the work conditions were the most critical factor for successful FIT implementation. Consistent with this finding, a report by Aiken et al. (2011) drawing upon 25 years of research in several countries, including Germany, showed that work conditions had positive impacts on nurse and patient outcomes (49). The relevant aspects of so called ‘work environment’ were operationalized by Aiken et al. as adequate staffing resources, nurse management ability and leadership, nurse-physician relations, nurse participation in hospital affairs, and the presence of nursing foundations for quality of care (49). Hospitals with consistently superior work environments had distinct advantages as: lower burnout rates for nurses, higher likelihood that nurses would report that their patients were ready for discharge, and lower probability of having nurses who were dissatisfied with their job, or who deemed the quality of care on their wards to be only fair or poor (49). Furthermore, in the context of psychiatry, positive aspects of organizational behavior such as unit manager’s skill at leadership, strong collegial nurse-physician relationships, and higher nurse-patient staffing ratios have all been associated with lesser occurrence of nurse burnout as well as lower rates of adverse clinical events (50–52).

These factors for successful FIT implementation show differences between the physicians’/psychologists’ and nurses’ experiences and evaluations. The main differences are highlighted in the following section.

### Differences Between Occupational Groups

Physicians and psychologists experienced FIT-related changes earlier in the implementation process and also more often than did nurses. Furthermore, nurses’ evaluations were less positive in every FIT-specific component. As suggested by our finding of the importance of project duration, physicians/psychologists became more easily accustomed to FIT-related changes than did nurses after a project duration of two or more years. We also see a (though not significant) trend toward worse evaluations from nurses with longer project duration and among nurses acting in a supervisorial role over other nurses. A sustained increase in workload, which is a plausible factor for additional stress for nurses during the implementation process, likely explains the greater importance of project duration as a factor for success perceived for physicians/psychologists. Since the experiences between the groups differed mainly for the component ‘expansion of professional expertise’ (which 20% of physicians/psychologists vs. 35% of nurses stated as “nonexistent”), we suppose that an unbalanced (re)distribution of tasks between the occupational groups during the implementation process may be a key reason for the differing ratings. Such a task redistribution was found by the study of Bartholomeyczik et al. (2008), where physicians seemingly passed on more tasks to nurses, while nurses were generally unable to reciprocate or engage other occupational groups (53). The degree of FIT implementation, as measured by EV, had the greatest difference between groups for the components ‘outreach home care’ and ‘multi-professional cooperation’. On the other hand, ‘shifting in- to outpatient setting’ showed the least difference between the evaluations of the groups. As several FIT departments did not implement outreach home care at the time of data collection, these discrepant experiences may arise from the physicians/psychologists being more involved in the theoretical aspects of new developments than were nurses, such that they had a better opportunity to understand and identify with this component (54). In contrast to ‘outreach home care’, a shift of treatment units from inpatient- towards day-patient and/or outpatient treatment setting was evident as the main component in ‘shifting in- to outpatient setting’ at the very onset of FIT projects (23). This shows that staff members with different occupational backgrounds had comparably positive identification with this component.

Although multi-professional cooperation was the component most strongly experienced by staff, the high discrepancy between staff evaluations indicates that divergent and possibly conflicting viewpoints may occur at the interface of occupational groups, which could certainly present a barrier for successful FIT implementation. Consistent with this finding, other studies have reported a persistent failure to attain adequate multi-professional cooperation (52, 55). A point of criticism in this regard is that a common understanding about objectives of patient care, extending beyond the simple label “patient-centered”, is often lacking (56). For example, medical and nursing processes often undergo separate planning, without addressing their mutual impacts and conditions. Specifically, there can be insufficient agreement about treatment objectives, which is compounded by the separate documentation systems for physicians and nurses (56, 57). Moreover, multi-professional cooperation mainly rests on the self-organization of wards/functional areas and such activities are typically regulated informally (55, 56).

### Strengths and Limitations of the Study

This is the first study judging the degree of FIT implementation based on psychiatric staff members’ experiences and evaluations of FIT in Germany. Therefore, the present results may inform about further adaptations necessary for improved FIT implementation on different organizational levels. Thus, results of the study may contribute to the development of national and international FIT projects.

According to our understanding, FIT is primarily a personal service. Therefore, we adopted a bottom-up strategy to measure the degree of implementation from the staff perspective, as indicated by the calculated values EX and EV. The strength of this strategy lies in its capacity to capture the perspective of those health workers who initiate and actively engage in treatment processes, or conversely those staff who (for whatever reason) present a barrier to implementation efforts. Therefore, gaining insight into the staff perspective plays a critical role for better understanding the factors underlying successful FIT implementation. Concurrent performance of staff surveys may also facilitate the organization’s ability to “unfreeze” and therefore obtain greater flexibility in creating readiness for implementation.

We note that the present study design may be vulnerable to some selection bias. Some staff might have refused to participate in the study because they are not interested in the implementation of the new treatment model or do not agree with its aims and implications. We cannot exclude the possibility that staff who support the model might have been over-represented in the group of survey respondents. Certainly, self-reporting brings a well-known risk of information bias (58). Staff who support FIT might have given more positive answers, while those with reservations may feel pressured to participate, or even be fearful of consequences despite the guarantee of their anonymity. Moreover, the key question to quantify EX and EV was too long and therefore might have caused difficulty in understanding as well as withdrawal from filling in the questionnaires.

As the present study was exploratory in nature, our findings need further validation in prospective studies. Our cross-sectional design limits the making of causal inferences and we can therefore make no statements about the reproducibility of the results in other settings. The four factors for successful FIT implementation together explained 49% of the variance of EV for physicians/psychologists and 34% for nurses, corresponding to a strong effect in both cases. However, there must remain other relevant factors yet to be identified. We also concede that the study lacks the perspective of special therapists, who were excluded from data analysis because of the considerable heterogeneity of their professional backgrounds and fields of activities. This may have decreased the transferability of present findings to other contexts.

### Practical Implications and Directions for the Future

Because attaining a high degree of implementation requires that a sufficient understanding of FIT-specific components ‘reaches’ or gets through to staff, it follows that closing the gap of experiences and evaluations between the occupational groups should be of high priority. Enabling this process would require a deeper integration of tasks as well as equal participation opportunities for the different occupational groups, e.g. entailing new forms of cooperation and training programs.

In the following section, we present practical implications and directions for the future based on present findings.

#### New Forms of Multi-Professional Cooperation

As early as 2007, the German Advisory Council on the Assessment of Developments in the Health Care System noted that the distribution of tasks between occupational groups did not meet the demographic, structural, and innovation-related requirements of the healthcare system (47). To close the gap of experience and evaluation between the occupational groups, FIT departments may have an opportunity systematically to develop and test new forms of multi-professional cooperation and competencies for occupational groups. These efforts could be tailored to the recommendations of the Advisory Council and the stated aims of FIT, which are well compatible and mutually beneficial. The Council recommended that new forms of cooperation should not primarily derive from the interests of any single occupational group, but from patient-based future demands of the healthcare system. However, it would be overly simplistic merely to (re)distribute single tasks within a system. Such an approach would likely increase the existing disparities between occupational groups on different levels. For example, disparities in workload on a micro-level or disparities in accounting resources on meso- or macro-levels, without bringing a corresponding expansion of expertise, which is itself an FIT-specific aspiration. Therefore, we should raise the question of what precisely should be the professional profile for occupational groups in FIT. Another question is what tasks may properly be redistributed to focus best on meeting the demands of the health system and improving multi-professional cooperation.

#### Training Programs

Another pathway to facilitate adoption of FIT may be to implement obligatory and ideally multi-professional training programs, as this emerged as the only common factor for successful implementation identified by both occupational groups in our study. While the diffusion of FIT-related information by implicit processes (for example, driven by hierarchical organizational structure or problematic power dynamics) resists short-term alteration, a strategy for dissemination of information about FIT might be implemented rapidly through training programs. This exposure could increase the experience of staff with FIT, which is an essential factor for attaining better understanding and identification with FIT-specific components.

Training programs may also give an opportunity for different occupational groups to consider FIT as a common project with matching of tasks according to defined and shared objectives of optimal patient care (47, 56). Although not an end in itself, multi-professional cooperation is a necessary precondition for attaining worthwhile interactions between all participants (17, 56). As other studies have shown, the implementation of a multi-professional treatment philosophy is not always free of conflicts, but presupposes an enhanced willingness of staff to negotiate amongst each other towards achieving a common goal (53, 55).

#### Systematic Change Management

As mentioned above, we found that individual demographic characteristics of staff (e.g. age, qualification) played a less important role in explaining the degree of implementation than did structural- and particular characteristics of FIT. This aspect presents an additional argument for implementing workshops, training programs, and other internal and/or external training opportunities such as Change Management. Systematic Change Management programs can promote the modification of organizational behavior, structures and procedures, as well as professional attitudes. It takes time for individuals to assimilate new forms of work and to change work routines, which may have been established and reinforced for years. Especially in the departure phase during the first two years of FIT implementation, it is necessary to manage restraining forces such as double routines and the risk of increased workload. In addition to training programs, other measures as for example Change Management are necessary to avoid excessive workload, rejection of FIT-specific components, and inner emigration when managing change. Only then can changes become a durable and accepted feature of the daily routine. Although arising from a different clinical area, we note the exemplary results for programs to reduce catheter-associated urinary tract infections, which have demonstrated the success of measures to facilitate adaption of change. Examples for key factors for success were repeated training and other measures such as audit and feedback, provision of electronic applications as reminders (59–62), as well as efforts to maintain and encourage positive changes, for example by a sustainability plan. Another exemplary case reported by Bartholomeyczik et al. (2008) suggests that forcing a clinical implementation without process-related restructuring may have positive/exonerative effects for one group (physicians in the Bartholomeyczik study), whereas others (nurses) may experience an increased burden without concomitant expansion of expertise (53).

#### Nurses Work Conditions

Facilitation of FIT implementation may benefit from attending to the factors that bring untoward structural strain especially for nurses during the implementation process. Personnel assessment and the tailored design of work conditions may affect not only the implementation of FIT, but also the outcomes for all stakeholders. For example, there is strong evidence for a significant association between lower patient-to-nurse ratios and lower patient mortality (63–65) and risk-adjusted mortality (66–69). Furthermore, nurse burnout and job dissatisfaction appeared to be barometers for patient satisfaction in those same hospitals (70). As mentioned above, psychiatry department organizational behavior is also associated with the level of adverse events (50, 51).

As we report above, nurses may tend to be less involved in the theoretical development of FIT and may therefore have less opportunity than physicians/psychologists for active participation in the implementation process. Therefore, we see a need for more brings the voice of nurses into FIT-related decisions, aiming to facilitate the implementation process and enable better outcomes for patients and nurses alike (49).

#### Further Research

Our exploratory findings need further substantiation and development to improve the practical implementation of FIT. Longitudinal observations over greater time intervals are necessary to support causal inferences and to enable the drawing of firm conclusions about the generalizability of the present results. In could be useful to implement a multilevel analysis or structural equation modelling approach in our analysis, as this approach might impart a better understanding of the variables arising from different organizational levels and their possible interplay. As individual characteristics of staff such as age and qualification seem to be minor factors for explaining EV, it would be interesting to survey the relevance of personal traits for successful implementation. Future research on FIT implementation should differentiate single work conditions such as supervision and the presence of hierarchy or cooperation among occupational groups, as well as the workload, participation opportunities, and (re)distribution of tasks between the occupational groups.

## Data Availability Statement

The datasets underlying the current study are not publicly available due to the used data protection declaration. Parts of the datasets are available from the corresponding author on reasonable request.

## Ethics Statement

The studies involving human participants were reviewed and approved by the Ethics Committee Brandenburg [2016, No. S 7 (a)], thus adhering to the ethical standards laid down in the 1964 Declaration of Helsinki and its later amendments. Written informed consent for participation was not required for this study in accordance with the national legislation and the institutional requirements.

## Author Contributions

SI wrote the first draft of the manuscript. SP and MH modified successive drafts. SI and BB developed the study design and were responsible for the statistical analysis and the interpretation of the data. All authors contributed to the article and approved the submitted version.

## Funding

Nine hospital government bodies pooled their resources to fund the study ‘EvaMod64b’ including the present study as declared under the section *Conflict of Interest*.

## Conflict of Interest

BB, SP, and MH received a financial grant for the study “EvaMod64b” from nine hospital governments bodies interested in the evaluation of their clinical FIT projects.

The remaining authors declare that the research was conducted in the absence of any commercial or financial relationships that could be construed as a potential conflict of interest.

The reviewer AB declared a shared affiliation, with no collaboration, with one of the authors, SI, to the handling editor at the time of review.

## References

[1] Dieterich M Irving CB Park B Marshall M Intensive case management for severe mental illness. Cochrane Database Syst Rev (2010) 10. 10.1002/14651858.CD007906.pub2 PMC4233116 20927766

[2] Johnson S Crisis resolution and home treatment teams: an evolving model. Adv Psychiatr Treat (2013) 19(2):115–23. 10.1192/apt.bp.107.004192

[3] Phillips SD Burns BJ Edgar ER Mueser KT Linkins KW Rosenheck RA Moving assertive community treatment into standard practice. Psychiatr Serv (2001) 52(6):771–9. 10.1176/appi.ps.52.6.771 11376224

[4] Stein LI Test M Alternative to mental hospital treatment: I. Conceptual model, treatment program, and clinical evaluation. Arch Gen Psychiatry (1980) 37(4):392–7. 10.1001/archpsyc.1980.01780170034003 7362425

[5] Caldas Almeida JM Mateus P Tomé G Joint action on mental health and well-being. Towards community-based and socially inclusive mental health care. Joint Action Ment Health Well-Being (2016) 152.

[6] Salize H Rössler W Becker T Mental health care in Germany: current state and trends. Eur Arch Psychiatry Clin Neurosci (2007) 257(2):92–103. 10.1007/s00406-006-0696-9 17149540

[7] Gijswijt-Hofstra M Oosterhuis H Vijselaar J Freeman H Psychiatric cultures compared: psychiatry and mental health care in the twentieth century: comparisons and approaches. Amsterdam: University Press (2005). 456p.

[8] Sachverständigenrat zur Begutachtung der Entwicklung im Gesundheitswesen: Koordination und Integration – Gesundheitsversorgung in einer Gesellschaft des längeren Lebens (2009). Sondergutachten. Available at: https://www.svr-gesundheit.de/fileadmin/user_upload/Gutachten/2009/Kurzfassung-2009.pdf (Accessed August 05, 2019). English short version (Coordination and Integration – Health Care in an Ageing Society). Available at: (Accessed August 05, 2019).

[9] Sachverständigenrat zur Begutachtung der Entwicklung im Gesundheitswesen: Wettbewerb an der Schnittstelle zwischen ambulanter und stationärer Gesundheitsversorgung (2012). Sondergutachten. Available at: https://www.svr-gesundheit.de/fileadmin/user_upload/Gutachten/2012/GA2012_Langfassung.pdf (Accessed August 05, 2019). English short version (Competition at the Interfaces between inpatient and outpatient Healthcare). Available at: https://www.svr-gesundheit.de/fileadmin/user_upload/Gutachten/2012/Kurzfassung-eng_formatiert.pd (Accessed August 05, 2019).

[10] Sachverständigenrat zur Begutachtung der Entwicklung im Gesundheitswesen: Bedarfsgerechte Steuerung der Gesundheitsversorgung. Gutachten (2018). Available at: https://www.svr-gesundheit.de/fileadmin/user_upload/Gutachten/2018/English_Summary_2018.pdf (Accessed August 05, 2019). English short summary (Needs-Based Regulation of the Health Care Provision). Available at: https://www.svr-gesundheit.de/fileadmin/user_upload/Gutachten/2018/English_Summary_2018.pdf (Accessed August 05, 2019).

[11] Gesetzliche Krankenversicherung. Sozialgesetzbuch (SGB V) Fünftes Buch. § 64b SGB V Modellvorhaben zur Versorgung psychisch kranker Menschen. (2018). Zuletzt geändert durch Art. 12 G v. 9.8.2019 I 1202. Available online at: https://www.sozialgesetzbuch-sgb.de/sgbv/64b.html (Accessed December 26, 2019).

[12] Deister A Vom Fall zum Menschen. Erfahrungen aus einem Regionalen Psychiatrie-Budget. Das Gesundheitswes (2011) 73(2):85–8. 10.1055/s-0030-1270493 21290353

[13] Roick C Heinrich S Deister A Zeichner D Birker T Schomerus G Das Regionale Psychiatriebudget: Kosten und Effekte eines neuen sektorenübergreifenden Finanzierungsmodells für die psychiatrische Versorgung. Psych Prax (2008) 35(6):279–85. 10.1055/s-2008-1067432 18773374

[14] Roick C Deister A Zeichner D Birker T König HH Angermeyer MC Das Regionale Psychiatriebudget: Ein neuer Ansatz zur effizienten Verknüpfung stationärer und ambulanter Versorgungsleistungen. PsychPrax (2005) 32(4):177–84. 10.1055/s-2004-834736 15852210

[15] British Medical Association (2018). Models for paying providers. Available at: https://www.bma.org.uk/collective-voice/policy-and-research/nhs-structure-and-delivery/nhs-structures-and-integration/models-for-paying-providers (Accessed September 01, 2019).

[16] Jacobs R Chalkley M Aragón MJ Böhnke JR Clark M Moran V Funding approaches for mental health services: is there still a role for clustering? B J Psych Adv (2018) 24(6):412–21. 10.1192/bja.2018.34 PMC6217930 30410789

[17] Deister A Wilms B Regionale Verantwortung übernehmen: Modellprojekte in Psychiatrie und Psychotherapie nach §64b SGB V. 1st ed. Köln: Psychiatrie Verlag (2014). p. 280.

[18] König HH Heinrich S Heider D Deister A Zeichner D Birker T The Regional Psychiatry Budget (RPB): A model for a new payment system of hospital based mental health care services? Psychiat Prax (2010) 37(1):34–42. 10.1055/s-0029-1223418 20072988

[19] Coleman M Schnapp W Hurwitz D Hedberg S Cabral L Laszlo A Overview of publicly funded managed behavioral health care. Adm Policy Ment Health Ment Health Serv Res (2005) 32(4):321–40. 10.1007/s10488-004-1662-3 15844852

[20] Mechanic D Seizing Opportunities Under the affordable care act for transforming the mental and behavioural health system. Health Aff (Millwood) (2012) 31(2):376–82. 10.1377/hlthaff.2011.0623 22323168

[21] Kliemt R Häckl D Neumann A Schmitt J Modellprojekte zur Versorgung psychisch kranker Menschen nach § 64b SGB V - Überblick und erste Ergebnisse der bundeseinheitlichen Evaluation. Auszug aus Barmer Gesundheitswes aktuell (2018) 156–79.

[22] Wasem J Reifferscheid A Südmersen C Faßbender R Thomas D (2012). Das pauschalierende Entgeltsystem für psychiatrische und psychosomatische Einrichtungen – Prüfung der Eignung alternativer Abrechnungseinheiten gemäß dem gesetzlichen Prüfauftrag nach § 17d Abs. 1 S. 2 KHG, IBES-Diskussionsbeitrag, Nr. 195, Fakultät Wirtschaftswissenschaften, Universität Duisburg-Essen, Essen [Preprint]. Available at: https://www.wiwi.uni-due.de/fileadmin/fileupload/WIWI/pdf/IBES_195_Gutachten_Psych-Entg_Final.pdf (Accessed August 05, 2019).

[23] von Peter S Ignatyev Y Johne J Indefrey S Kankaya OA Rehr B Evaluation of flexible and integrative psychiatric treatment models in Germany - A mixed-method patient and staff-oriented exploratory study. Front Psychiatry (2019) 9:785. 10.3389/fpsyt.2018.00785 30723433 PMC6349706

[24] Johne J von Peter S Schwarz J Timm J Heinze M Ignatyev Y Evaluation of new flexible and integrative psychiatric treatment models in Germany- assessment and preliminary validation of specific program components. BMC Psychiatry (2018) 18:278. 10.1186/s12888-018-1861-1 30176836 PMC6122621

[25] Böhle F Interaktionsarbeit als wichtige Arbeitstätigkeit im Dienstleistungssektor. WSI Mitteilungen (2011) p. 456–61 https://www.boeckler.de/data/wsimit_2011_09_boehle.pdf.

[26] Dunkel W (2002). Interaktive Arbeit und personenbezogene Dienstleistungen. Thesen zu konzeptionellen und empirischen Defiziten der Arbeitsforschung. [Preprint]. Available at: https://www.isf-muenchen.de/pdf/kickoff_paper_wd.pdf (Accessed August 31, 2019).

[27] Gross P Badura B Sozialpolitik und soziale Dienste: Entwurf einer Theorie personenbezogener Dienstleistungen. In: Von Ferber C Kaufmann FX, editors. Soziologie und Sozialpolitik (Sonderheft 19 der Kölner Zeitschrift für Soziologie und Sozialpsychologie). Opladen: Westdeutscher Verlag (1977). p. 375–405.

[28] Ferlie EB Shortell SM Improving the quality of health care in the United Kingdom and the United States: a framework for change. Milbank Q (2001) 79(2):281–315. 10.1111/1468-0009.00206 11439467 PMC2751188

[29] Schein EH Process consultation revisited: Building the helping relationship. Boston, Massachusetts, USA: Reading, Addison Wesley Longman (1999). p. 272.

[30] Miller VD Johnson JR Grau J Antecedents to willingness to participate in a planned organizational change. J Appl Commun Res (1994) 22(1):59–80. 10.1080/00909889409365387

[31] Wanberg CR Banas JT Predictors and outcomes of openness to change in a reorganizing workplace. J Appl Psychol (2000) 85(1):132–42. 10.I037//0021-9010.85.1.132 10740964

[32] Cummings TG Worley G Organization development and change. Cincinnati, OH: South-Western Publishing (2009). p. 772.

[33] Greenhalgh T Robert G Macfarlane F Bate P Kyriakidou O Diffusion of innovations in service organizations: systematic review and recommendations. Milbank Q (2004) 82(4):581–629. 10.1111/j.0887-378X.2004.00325.x 15595944 PMC2690184

[34] Jones RA Jimmieson NL Griffiths A The impact of organizational culture and reshaping capabilities on change implementation success: The mediating role of readiness for change. J Manage Stud (2005) 42(2):361–86. 10.1111/j.1467-6486.2005.00500.x

[35] Mayntz R Implementation politischer Programme II: Ansätze zur Theoriebildung. Opladen: Verlag für Sozialwissenschaften (1983). p. 256.

[36] Von Peter S Ignatyev Y Indefrey S Johne J Schwarz J Timm J Specific components for integrative and flexible care models according to § 64b SGB V. Der Nervenarzt (2017) 89:559–64. 10.1007/s00115-017-0459-z 29209751

[37] Fischbeck S Laubach W Arbeitssituation und Mitarbeiterzufriedenheit in einem Universitätsklinikum: Entwicklung von Messinstrumenten für ärztliches und pflegerisches Personal. Psychother Med Psychol (2005) 55(6):305–14. 10.1055/s-2004-834753 15948051

[38] Laubach W Milch W Ernst R Dimensionen der Arbeitsbelastung und Arbeitszufriedenheit in der psychiatrisch-psychotherapeutischen Pflege. Psychother Psychosom Med Psychol (1999) 49:37–48. 10098391

[39] Milch W Ernst R Laubach W Kooperation im pflegerisch-ärztlichen Team. Eine Analyse pflegerischer Bewertungen in der psychiatrischen Pflege. Psychiat Prax (1999) 26(3):122–7. 10412708

[40] Zander B Dobler L Bäumler M Busse R Nursing tasks left undone in german acute care hospitals – results from the international study RN4Cast. Gesundheitswesen (2014) 76:727–34. 10.1055/s-0033-1364016 24771101

[41] Cronbach LJ Coefficient alpha and the internal structure of tests. Psychometrika (1951) 16:297–334. 10.1007/BF02310555

[42] Cohen J Statistical Power Analysis for the behavioral sciences. 2. ed. Hillsdale: Lawrence Erlbaum Associates (1988). p. 400.

[43] Brady GP Cummings GG The influence of nursing leadership on nurse performance: a systematic literature review. J Nurs Manag (2010) 18(4):425–39. 10.1111/j.1365-2834.2010.01100.x 20609047

[44] McGoldrick TB Menschner EF Pollock ML Nurturing the transformation from staff nurse to leader. Holist Nurs Pract (2001) 16(1):16–20. 10.1097/00004650-200110000-00006 15559043

[45] Stemmer R Zur Situation der Pflege im Krankenhaus. Pflege Gesellschaft (2011) 16(4):293–303.

[46] Zangaro GA Soeken KL A meta-analysis of studies of nurses’ job satisfaction. Res Nurs Health (2007) 30(4):445–58. 10.1002/nur.20202 17654483

[47] Sachverständigenrat zur Begutachtung der Entwicklung im Gesundheitswesen: Kooperation und Verantwortung – Voraussetzungen einer zielorientierten Gesundheitsversorgung (2007). Available at: https://www.svr-gesundheit.de/fileadmin/user_upload/Gutachten/2007/Kurzfassung_2007.pdf (Accessed August 05, 2019). English short version (Cooperation and Responsibility - Prerequisites for Target-Oriented Health Care). Available at: https://www.svr-gesundheit.de/fileadmin/user_upload/Gutachten/2007/KF2007-engl.pdf (Accessed August 05, 2019).

[48] Nelson T Johnson S Bebbington P Satisfaction and burnout among staff of crisis resolution, assertive outreach and community mental health teams: A multicenter cross sectional survey. Soc Psychiatry Psychiatr Epidemiol (2009) 44(7):541–9. 10.1007/s00127-008-0480-4 19082906

[49] Aiken LH Sloane DM Clarke S Poghosyan L Cho E You L Importance of work environments on hospital outcomes in nine countries. Int J Qual Health Care (2011) 23(4):357–64. 10.1093/intqhc/mzr022 PMC3136199 21561979

[50] Hanrahan NP Aiken LH McClaine L Hanlon AL Relationship between psychiatric nurse work environments and nurse burnout in acute care general hospitals. Issues Ment Health Nurs (2010a) 31(3):198–207. 10.3109/01612840903200068 20144031 PMC2856615

[51] Hanrahan NP Kumar A Aiken LH Adverse events associated with organizational factors of general hospital inpatient psychiatric care environments. Psychiatr Serv (2010b) 61(6):569–74. 10.1176/ps.2010.61.6.569 PMC2890256 20513679

[52] Gabrielsson S Looi GME Zingmark K Sävenstedt S Knowledge of the patient as decision-making power: staff members’ perceptions of interprofessional collaboration in challenging situations in psychiatric inpatient care. Scand J Caring Sci (2014) 28:784–92. 10.1111/scs.12111 24400837

[53] Bartholomeyczik S Donath E Schmidt S Rieger MA Berger E Wittich A Arbeitsbedingungen im Krankenhaus. Abschlussbericht zum Projekt “Arbeitsbedingungen im Krankenhaus”, Projekt F 2032. Dortmund, Berlin, Dresden: Im Auftrag der Bundesanstalt für Arbeitsschutz und Arbeitsmedizin (2008). Available at: https://www.baua.de/DE/Angebote/Publikationen/Berichte/F2032.pdf?__blob=publicationFile (Accessed December 23, 2019).

[54] Drake RE Essock SM Shaner A Karey KB Minkoff K Kola L Implementing dual diagnosis services for clients with severe mental illness. Psychiatr Serv (2001) 52:469–76. 10.1176/appi.ps.52.4.469 11274491

[55] Zwack J Schweitzer J Multiprofessionelle systemisch–familientherapeutische Teamweiterbildung in der Akutpsychiatrie Auswirkungen auf die Teamkooperation und die Mitarbeiterbelastung [Multiprofessional Family–System Training Programme in Psychiatry. Effects on Team Cooperation and Staff Strain]. Psychiat Prax (2008) 35:15–20. 10.1055/s-2006-952028 17594634

[56] Dahlgaard K Verbesserung der teamorientierten Zusammenarbeit zwischen Ärzten und Pflegenden – Neue Chancen durch Prozessorientierung und erweiterte Aufgaben für Pflegende. Z Evid Fortbild Qual Gesundh wesen (ZEFQ) (2010) 104:32–8. 10.1016/j.zefq.2009.12.004 20369443

[57] Tschinke I Effektive Prozessgestaltung in der interdisziplinären Teamarbeit. Psych Pflege (2004) 10(4):203–7. 10.1055/s-2004-813074

[58] Van de Mortel T Faking it: social desirability response bias in self-report research. Aust J Adv Nurs (2008) 25(4):40–8.

[59] Bell MM Alaestante G Finch C A Multidisciplinary intervention to prevent catheter-associated urinary tract infections using education, continuum of care, and systemwide buy-in. Ochsner J (2016) 16(1):96–100. 27046414 PMC4795513

[60] Agency for Healthcare Research and Quality Guide to implementing a program to reduce catheter-associated urinary tract infections in long-term care. AHRQ Publication No. 16(17)-0003-5-EF, March 2017. Available at: https://www.ahrq.gov/hai/quality/tools/cauti-ltc/modules/implementation/guide.html (Accessed December 27, 2019).

[61] Mauger B Marbella A Pines E Chopra R Black ER Aronson N Implementing quality improvement strategies to reduce healthcare-associated infections: A systematic review. Am J Infect Control (2014) 42(10 Suppl):274–83. 10.1016/j.ajic.2014.05.031 25239722

[62] Meddings J Rogers MA Krein SL Fakih MG Olmsted RN Saint S Reducing unnecessary urinary catheter use and other strategies to prevent catheter-associated urinary tract infection: an integrative review. BMJ Qual Saf (2014) 23(4):277–89. 10.1136/bmjqs-2012-001774 PMC3960353 24077850

[63] Kane RL Shamliyan T Mueller C Duval S Wilt T The association of registered nurse staffing levels and patient outcomes systematic review and meta-analysis. Med Care (2007) 45(12):1195–204. 10.1097/MLR.0b013e3181468ca3 18007170

[64] Shekelle PG Nurse–patient ratios as a patient safety strategy: a systematic review. Ann Int Med (2013) 158(5 Pt 2):404–09. 10.7326/0003-4819-158-5-201303051-00007 23460097

[65] Aiken LH Cerón C Simonetti M Lake ET Galiano A Garbarini A Hospital nurse staffing and patient outcomes. Rev Med Clin Condes (2018) 29(3):322–7. 10.1016/j.rmclc.2018.04.011

[66] Aiken LH Sloane DM Bruyneel L Van den Heede K Griffiths P Busse R Nurse staffing and education and hospital mortality in nine European countries: a retrospective observational study. Lancet (2014) 383(9931):1824–30. 10.1016/S0140-6736(13)62631-8 PMC4035380 24581683

[67] Aiken LH Clarke SP Sloane DM Sochalski J Silber JH Hospital nurse staffing and patient mortality, nurse burnout, and job dissatisfaction. JAMA (2002) 288(16):1987–93. 10.1001/jama.288.16.1987 12387650

[68] Estabrooks CA Midodzi WK Cummings GG Ricker KL Giovannetti P The impact of hospital nursing characteristics on 30-day mortality. Nurs Res (2005) 54(2):74–84. 10.1097/00006199-200503000-00002 15778649

[69] Rafferty AM Clarke SP Coles J Ball J James P McKee M Outcomes of variation in hospital nurse staffing in English hospitals: cross-sectional analysis of survey data and discharge records. Int J Nurs Stud (2007) 44(2):175–82. 10.1016/j.ijnurstu.2006.08.003 PMC2894580 17064706

[70] McHugh MD Kutney-Lee A Cimiotti JP Sloane DM Aiken LH Nurses’ widespread job dissatisfaction, burnout, and frustration with health benefits signal problems for patient care. Health Aff (Millwood) (2011) 30(2):202–10. 10.1377/hlthaff.2010.0100 PMC3201822 21289340

